# Learning from soil gas change and isotopic signatures during 2012 Emilia seismic sequence

**DOI:** 10.1038/s41598-017-14500-y

**Published:** 2017-10-27

**Authors:** Alessandra Sciarra, Barbara Cantucci, Massimo Coltorti

**Affiliations:** 1 Istituto Nazionale di Geofisica e Vulcanologia, sezione di Sismologia e Tettonofisica, via di Vigna Murata 605, 00143 Rome, Italy; 2 Department of Physics and Earth Sciences University of Ferrara, Ferrara, Italy; 3 Consiglio Nazionale delle Ricerche – Istituto di Geologia Ambientale e Geoingegneria, Rome, Italy

## Abstract

Soil surveys were performed in Medolla (Italy), a peculiar area characterized by spotty high soil temperature, gas vent, and lack of vegetation, to determine the migration mechanisms and spatial behavior of gas species. Hereby we present soil gas measurements and their isotopic ratios measured between 2008 and 2015, including the 2012 Emilia-Romagna seismic sequence. We found that soil gas concentrations markedly changed during the main shocks of May 20 and 29, 2012 (Mw 6.1 and 6.0, respectively), highlighting the presence of a buried fault intersecting the gas vents. We suggest that crustal dilation associated with seismic activity favored the uprising of geogas towards the surface. Changes in the isotopic signature highlight the contribution of two distinct sources, one deeper, thermogenic and another superficial related to organic-rich layer, whose relative contribution varied before, during and after the earthquake. We suppose an increase of microbial component likely due to the ground shaking of shallower layers linked to seismic sequence, which masks the thermogenic contribution. Although the changes we detect are specific for an alluvial plain, we deduce that analogous processes may be active elsewhere, and that soil gas geochemistry represents an useful tool to discriminate the gas migration related to seismic activity.

## Introduction

Soil gas geochemistry in seismically active areas has been widely used to localize buried faults and co-seismic fractures^1–5^. The stress/strain changes related to seismic activity may in fact force crustal fluid to migrate upward, especially along active faults, thus altering the geochemical spatial distribution of the soil gas^6,7^.

Most soil gas surveys, however, are typically carried out after the main earthquake event/s, precluding the possibility to evaluate the stress/strain effect on gas species before and after the event. Only a little information is thus available on fluid activity and circulation during a pre-seismic period.

Hereby we present 37 soil gas concentrations (CO_2_, CH_4_, H_2_, He, Ne, C_2_H_6_, ^222^Rn) together with the isotopic ratios of CH_4_ and CO_2_ (δ^13^C-CH_4_, δ^13^C-CO_2_, δD-CH_4_) that were periodically measured in the period 2008–2015 and immediately after the 2012 Emilia seismic sequences.

**Figure 1:**
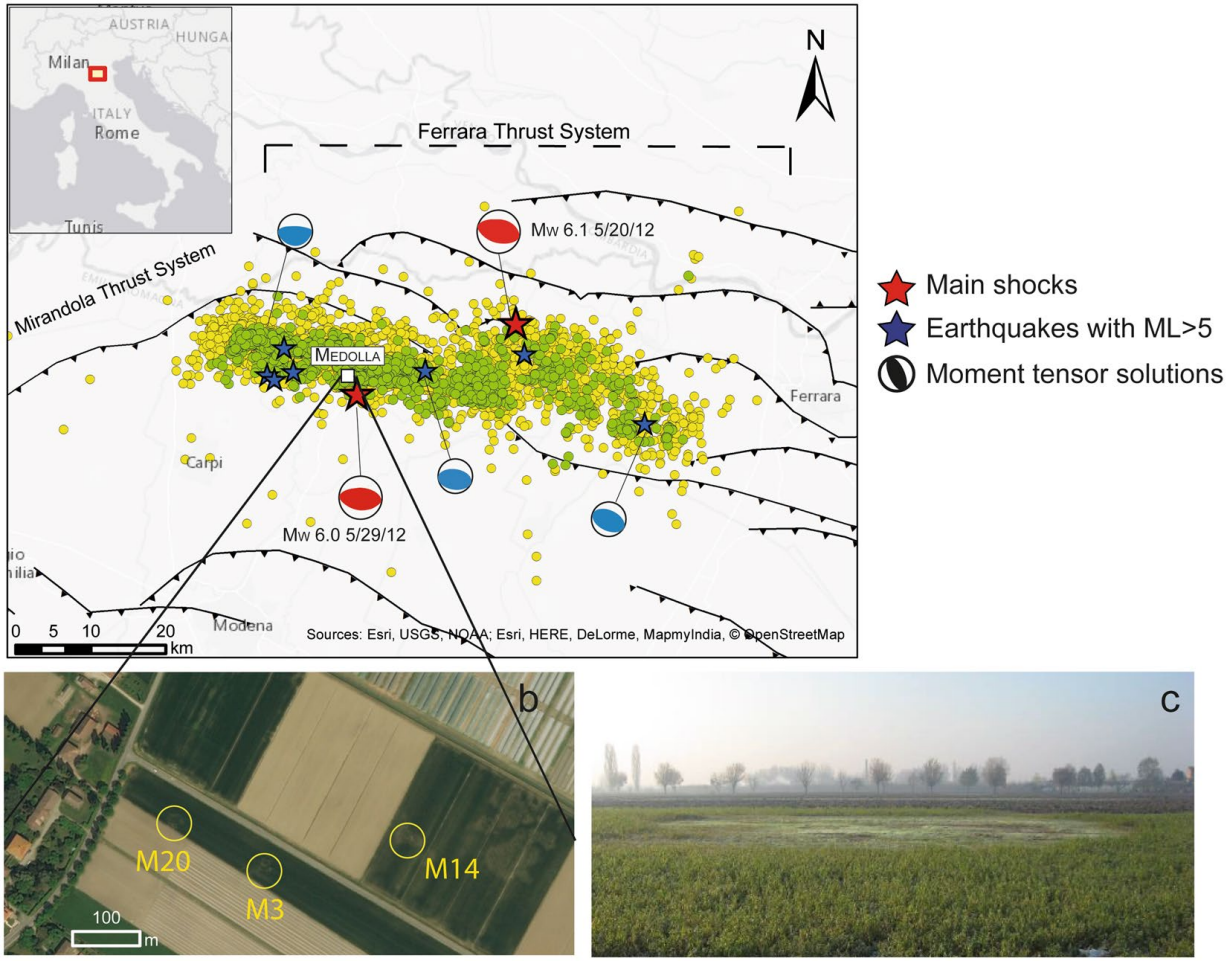
(**a**) Seismotectonic framework of the study area. Solid black lines represent the major active thrust faults of the area^18,36^. The orange circles are the relocated aftershocks of the first year after the two main shocks^37^, while the green circles are 3-D relocated aftershocks of the first month of the sequence^18^ [this figure has been constructed using Esri ArcGIS ArcMap 10.2.1 http://www.esri.com/software/arcgis/arcgisfor-desktop for Desktop]. (**b**) Satellite imagery of the study area with evidence of M20, M3, and M14 macroseeps [basemap was obtained from Esri ArcGIS ArcMap 10.2.1]. (**c**) Landscape picture showing extension of a macroseep, delineated by the absence of vegetation.

Measurements were performed near Medolla (a small municipality in the Emilia-Romagna Region, Italy; Fig. 1a), in a farming area less than 1 km^2^ wide, where the presence of a remarkably high temperature of the soils (up to 48.5 °C) associated with methane seepage, has been known since 1893^8^. In some cases, this seepage phenomenon is highlighted by the occurrence of several subcircular areas (<10 m in diameter) where crops and vegetation are unable to grow (Fig. 1b,c) and during wintertime as the snow cover quickly melts. The location of the main macroseep area has remained relatively stable over the past 120 years, although with different intensity.

Recent studies^9,10^ suggested that the anomalous ground heating is not linked with local ascents of hot fluids from depth, but could be the result of exothermic oxidation of shallow (<1m) biogenic methane (δ^13^C-CH_4_ ranging from −62.5‰ to −72.3‰ VPDB), enhanced by the activity of methanotrophic bacteria^11^.

The investigated site lies on the top of the central portion of the Ferrara arc (Fig. 1a), which represents the external fold-and-thrust system of the Northern Apennines thrust belt. The Ferrara arc consists of two major blind thrust systems, the Ferrara Thrust System to the northeast and the Mirandola Thrust System to the west, and contains a thick and folded sedimentary succession mainly made up of Triassic evaporites, Jurassic-Cretaceous carbonates and Oligocene-Miocene clastic deposits covered by Plio-Pleistocene sandy turbidites and Late Quaternary fluvio-lacustrine deposits of the Po plain^12,13^. This sector of the buried Apennine front is tectonically active in response to the general compressive stress field^14^ dating from middle Pleistocene.

In the Medolla area, natural gas (CH_4_-dominated) reservoirs have been detected at depths between 2000 and 2700 m, in correspondence of reverse fault planes^15^ from which fluids migrate upward along minor fault planes. At these depths, the main stratigraphic unit consists of upper Miocene marls and organic-rich clays^15^. Moreover, some other CH_4_-dominated gas occurrences are recognized at 200 m and between 650 and 900 m of depth in the Plio-Pleistocene formations^16^. According to Lindquist^16^ and Mattavelli and Novelli^17^ most of the gaseous hydrocarbons in the Po Plain have a biogenic origin (80%), while the remaining is equally distributed between thermogenic (10%) and mixed origin (10%).

In May-June 2012, a seismic sequence struck the Emilia-Romagna Region, with more than 2,400 aftershocks. The epicenters of the two main shocks, Mw 6.1 and 6.0^18^ were located 15 and 2 km from the study area, respectively (Fig. 1a).

## Results

The soil gas surveys were carried out in October 2008, December 2008, soon after the first main-shock, in September 2012 and in the following years (2013, 2014 e 2015), aiming at monitoring any variations in the gas chemistry, as well as in the seepage spatial distribution. Several gas species (CO_2_, CH_4_, H_2_, He, Ne, C_2_H_6_, ^222^Rn) were determined, but in the following only CO_2_, H_2_ and CH_4_ measurements are reported, since they are the only gases where isotopic ratios were also analyzed.

**Figure 2:**
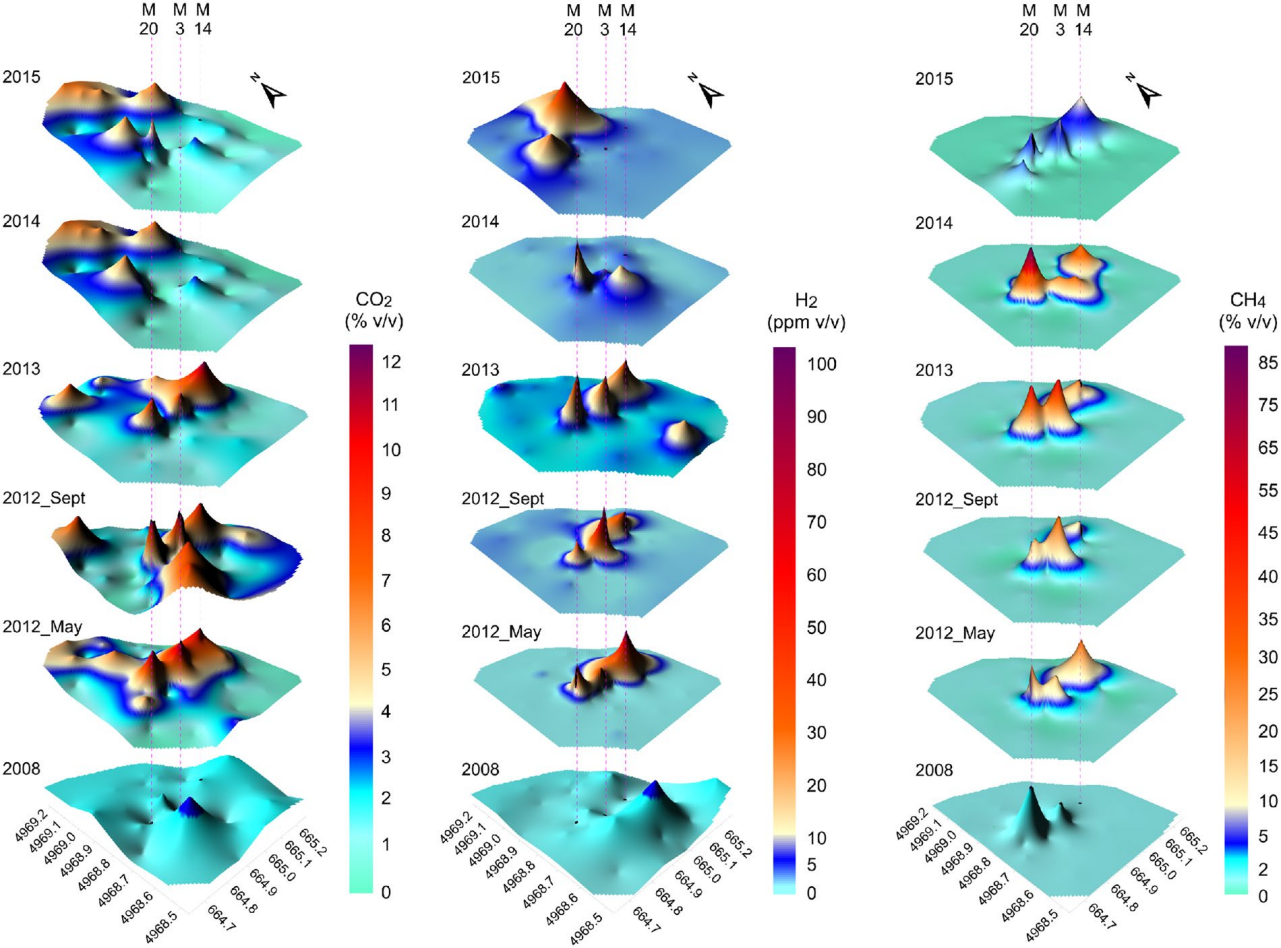
3D-contour maps of CO_2_, H_2_ and CH_4_ concentrations from October 2008 to May 2015. Dashed lines: projection of M20, M3, M14 macroseeps locations.

Collected data were processed with a standard statistical approach (Supplementary Table S1) and used to create three-dimensional surface maps (Fig. 2).

Normal Probability Plot (NPP) were used to select background, anomalous values, and extra outliers^19^. In particular values above 2.00% v/v for CO_2_, 1.8 ppm v/v for H_2_ and 0.10% v/v for CH_4_ have been considered as “anomalies”.

In October 2008 the investigated area was characterized by CO_2_, H_2_ and CH_4_ concentrations with average values of 0.13% v/v, 1.63 ppm v/v and 3.6 × 10^−3^% v/v, respectively. If compared with the data collected in 2006, 7 km southeastward from Medolla^20^, gas emissions exhibited much higher values of CH_4_ and H_2_, whereas CO_2_ was more than one order of magnitude lower (CO_2_, 2.31% v/v; H_2_, 0.44 ppm v/v; CH_4_, 6.01 × 10^−4^% v/v). The highest CH_4_ concentrations were measured in two areas with the absence of vegetation (0.048% v/v at M20 and 0.024% v/v at M3). It is likely that these macroseeps represent preferential migration pathways for deep gas hosted in the Mesozoic formations (>3000 m depth; Camurana 2 well log), as suggested by the δ^13^C-CH_4_ measured on these points showing thermogenic values of −25.88 and −29.68‰.

Soon after the main shock on 20^th^ of May 2012, CH_4_ average concentrations increased by more than three orders of magnitude (6.46% v/v), whereas H_2_ and CO_2_ showed an increment to 9.36 ppm v/v and 5.43% v/v respectively. The highest values of all gas species were observed at M20 and M3 (up to 39.00% v/v and 89.42 ppm v/v for CH_4_ and H_2_ at M20, and up to 13.50% v/v for CO_2_ at M3) and in a newly formed area with lack of vegetation (M14, 11.83% v/v, 86.56 ppm v/v and 40.34% v/v for CO_2_, H_2_ and CH_4_, respectively).

A few months later, in September 2012, average H_2_ concentrations increased further (12.28 ppm v/v), whereas CH_4_ and CO_2_ concentrations remained substantially stables, with the highest values (39.80% v/v and 12.25% v/v, respectively) in correspondence of M3 site. Some CO_2_ anomalous spots (with values ranging from 8.41% v/v to 8.94% v/v) were measured outside the macroseeps zone, in the northern and southern part of the study area. In absence of hints for a deep origin (as the association with other geogas) these anomalies can be attributed to organic material oxidation, microorganism or plant respiration^21^.

Between 2013 and 2014 the CH_4_ mean concentrations increased up to 7.53% v/v (highest value of 84.20% v/v at M20), whereas H_2_ decreased to 3.64 ppm v/v with the highest value of 37.2 ppm v/v always at M20. Mean CO_2_ concentrations decreased to 2.72% v/v (maximum value of 11.01% v/v on M3).

Finally, in 2015 the average concentrations of all gases dropped to 2.03% v/v, 4.61 ppm v/v and 0.70% v/v for CO_2_, H_2_ and CH_4_, respectively, remaining overall higher than the values collected in 2008. CH_4_ highest values were found on macroseeps (maximum value of 8.97% v/v on M14), whereas the highest CO_2_ and H_2_ values (6.32% v/v and 30.7 ppm v/v) were located in the northern and eastern part of the studied area.

The origin of soil gas was investigated by isotopic ratios of CH_4_ and CO_2_ measured on the points with highest concentrations (Table S2). As already mentioned δ^13^C-CH_4_, δD-CH_4_ measured on M3 and M20 in October ranged from −29.86‰ to −25.88‰ vs VPDB and −92.26‰ to −106.44‰ vs VSMOW, respectively. A few months later (December 2008) these points sensibly changed their CH_4_ isotopic ratios moving toward more negative values (ranging from −66.89‰ to −68.07‰ vs VPDB, and from −182.7‰ to −187.95‰ vs VSMOW), with only minor variations between 2012 and 2015. Other samples with anomalous concentrations showed substantially consistent δ^13^C-CH_4_, δD-CH_4_ values over the time, ranging from −21.51‰ to −78.85‰ vs VPDB and from −72.83‰ to −187.8‰ vs VSMOW.

δ^13^C-CO_2_, measured from December 2008 to May 2015, displayed a wide variation, from −10.96‰ up to −70‰ vs VPDB.

## Discussion

The anomalous concentration of CO_2_, H_2_ and CH_4_, highlighted a general E-W trend connecting the main macroseeps (Fig. 2). The association of more than one gas species along a linear trend suggests the presence of a previously unknown blind fault, similarly to what observed in other seismically active areas^4^. In soils, the average H_2_ concentration is about 0.5 ppm v/v, almost the same as that found in the atmosphere. High H_2_ concentrations, up to several thousand ppm, are restricted to active faults^22^. CH_4_ and H_2_ concentrations are positively correlated over time, with a Pearson coefficient ranging from 0.66 to 0.86. This relationship suggest that CH_4_ can act as a carrier for H_2_ which would be otherwise unable to reach the surface due to its low concentration^2,22^.

No evidence for this linear trend were observed before 2012, thus it can be inferred that seismic activity has altered stress field enhancing a pre-existent phenomenon, such as the gas seepage through the soil. Earthquakes and crustal deformation^4^ can indeed alter the hydraulic properties of soils, such as permeability and porosity, favoring advective migration of deep gases toward the surface caused by variations in pressure and temperature following preferential pathways.

In a low permeability soil, such as the Plio-Pleistocene sediments of the area, the overpressure generated by an earthquake can remain active for a long period and the effects on deformations and on fluid flow could be visible after several months^23^. The significant decrease of soil gas concentrations in 2015 may be due to both a reduction of permeability and porosity of rocks and soils in the rupture zone and a closure of the pathways opened by the seismic activity after the overpressure generated by the earthquake had reduced.

**Figure 3:**
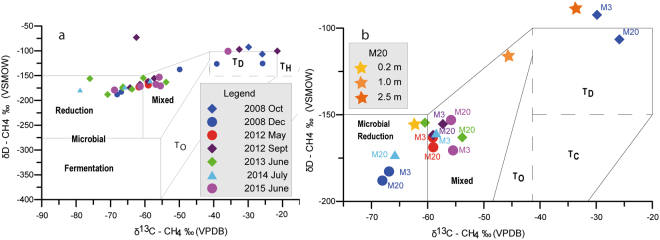
Methane carbon and hydrogen isotope diagram (genetic zonation revised and updated by ref.^38^, after ref.^24^). (**a**) all collected isotopic data; (**b**) zoom of the plot highlighting only the M3 and 20 data, and the vertical profile on M20 (0.2, 1.0, 2.5 m depth). TO, thermogenic with oil; TC, thermogenic with condensate; TD, dry thermogenic; TH: thermogenic with high-temperature CO_2_–CH_4_ equilibration.

Concerning the isotopic ratios (δ^13^C-CH_4_, δD-CH_4_ and δ^13^C-CO_2_) of soil gas, the traditional Shoell’s plot^24^ (Fig. 3a) highlights two different groups of data: one with prevailing microbial and mixed origin, and a few points falling in the thermogenic (TD) field. Thermogenic signature is positively correlated with low CH_4_ concentrations, whereas for increasing CH_4_ concentrations microbial and mixed sources prevail.

Overall, during the survey record, the isotopic signatures of most samples remained substantially consistent. However the most active M3 and M20 macroseeps switched their signature from thermogenic (October 2008) to both microbial and mixed origin (December 2008–2015; Fig. 3b). We suggest that in this area two different sources of methane coexist, one thermogenic, deriving from the deeper Mesozoic reservoirs^17^ (>3000 m), and another microbial^25^ produced at a shallower depth (between 200 and 900 m). It is likely that the relative increase of microbial component masks the thermogenic contribution. The lack of an evolutionary trend between thermogenic and mixed-microbial origin suggest that a fractioning process does not occur. On the contrary, the shift between mixed and microbial origin suggests the presence of a variable contribution of shallow input over time. A vertical profile, performed in 2015 at the M20 site (Fig. 3b; Supplementary Table S3) to verify this hypothesis, showed increasing thermogenic contribution with depth, shifting from microbial (0.20 m), to mixed (1 m), to thermogenic (2.5 m).

Samples with thermogenic origin showed δ^13^C-CO_2_ values (from −10.96‰ to −37.04‰) typical of organic and/or soil-derived origin^26^. On the contrary, extremely negative values, between −42.15 and −70.01‰, were recorded on samples with CH_4_ microbial and mixed origin. These negative values have the same^13^C/^12^C ratio of CH_4_, suggesting that CO_2_ directly derives from microbial CH_4_.

This hypothesis is consistent with Galand *et al*.^27^, Valentine *et al*.^28^ and Whiticar^29^ which showed that processes as bacterial methanogenesis may produce a carbon isotopic fractionation between CH_4_ and the coexisting CO_2_ ranging from 41‰ to 72‰, 22‰ to 58‰, and 49‰ to 95‰, respectively. These ranges are higher than the isotopic difference between CH_4_ and CO_2_ detected at Medolla area (from 28‰ to −2‰; Table S2). In fact, the depletion of ^13^C in CO_2_ is more compatible with a ^13^C/^12^C kinetic fractionation due to a partial CH_4_ to CO_2_ conversion which occur in the presence of free oxygen and methanotrophic bacteria^9–11^. Indeed, according to geochemical and biological data from Capaccioni *et al*.^9^ and Cappelletti *et al*.^11^, anomalous ground heating of Medolla area is due to oxidative conditions and bacterial activity (genus Methylocaldum), promoting the exothermic oxidation of methane within the most aerated soil layer at 0.6 and 0.7m depths^9^. This exothermic reaction (800 kJ × mol^−1^)^30^ could also explain the anomalous soil heating measured on macroseeps (from 30 to 48 °C) in the 2012–2015 period. This process had always been active over the time, although with different intensity, and could explain both the observed ground heating up, and the diffuse emission from the soil of CO_2_ characterized by an extremely negative isotopic (^13^C/^12^C) signature.

In this study, soil temperatures measured in other parts of the investigated area, showed average values of 24 °C consistent with air temperatures and regional geothermal gradient (1 °C/100 m)^31^, supporting the theory of CH_4_ oxidation as the main process for macroseeps soil heating.

Although the soil heating mechanism is independent from tectonic activity we suggest that 2012 seismic sequence might have enhanced this phenomenon increasing the preexisting CH_4_ emissions. Indeed the highest soil temperature (51.8 °C) was measured in May 2012 together with the highest CO_2_ and CH_4_ concentrations (11.7% v/v and 38.8% v/v, respectively). On the other hand when a gas uprising is high, CH_4_ is not completely consumed by bacteria^32^. According to the prevailing local conditions, some fraction of methane may escape oxidation reaching the surface.

## Conclusions

Soil gas distribution and their isotopic signature were investigated in the Medolla farming area, between 2008 and 2015. After the 2012 seismic sequence the soil gas concentrations of CO_2_, H_2_ and CH_4_ markedly increased along an E-W preferential direction, suggesting the presence of a buried tectonic lineament linking CH_4_ macroseeps. Seismic crustal deformation favored the fluid migration towards the surface by increasing the pore pressure and/or enhancing permeability of soils following preferential pathways. In 2015, these concentrations gradually decrease towards the initial values, although they remain still higher than those observed in 2008. This decreasing is likely due to a natural lowering of permeability and porosity in the rupture zone after the overpressure generated by the earthquake had reduced.

Isotopic ratios of CH_4_ and CO_2_ highlight two different gas sources, one deeper, thermogenic and another shallower, microbial. These sources coexist producing a variable isotopic ^13^C/^12^C ratio from microbial to mixed, depending on the contributions of each source. The lack of an evolutionary trend between thermogenic and mixed-microbial origin suggest that a fractioning process does not occur.

The extremely negative values of δ^13^C-CO_2_, recorded on samples with CH_4_ microbial and mixed origin, and the high soil temperatures are ascribed to the exothermic oxidative reactions of CH_4_ in CO_2_ which occur in the presence of free oxygen and methanotrophic bacteria. The macroseeps CH_4_ emission and the high soil temperature have been known since the late nineteenth century and are therefore independent from tectonic activity of 2012. The earthquakes might have enhanced the gas seepage phenomenon, favoring the uprising of microbial and mixed CH_4_. According to isotopic results, the ground shaking linked to the 2012 seismic sequence enhanced the migration of soil gases from the shallower layers of Plio-Pleistocene deposits, increasing the microbial contribution of methane and covering the low amount of deeper thermogenic gases.

Soil gas geochemistry represents an useful tool to discriminate the gas migration related to seismic activity. The long term geochemical monitoring allowed to recognize that after an initial variation of soil gas distribution linked to seismic activity, the system in the Medolla area is slowly returning to its pre-seismic condition. Obtained results encourage the research about soil gas geochemistry on seismic active area highlight the importance to have a dataset before, during and after earthquakes.

## Method

### Sampling procedure

37 soil gas samples were collected on a yearly basis from October 2008, except for two surveys in 2012. All surveys were conducted during a period of stable and dry weather conditions and in a short time to minimize any variations induced by different sampling periods. Samples were collected from the unsaturated or vadose zone using a steel probe driven into the ground to a depth of 0.8 m; this depth is considered to be below the major influence of meteorological variables^33,34^.

### Chemical analysis

The soil–gas concentrations (N_2_, O_2_, CO_2_, CH_4_, He, C_2_H_6_, H_2_) were analyzed in the Fluid Geochemistry Laboratory at INGV Rome, by a MicroGC Agilent 4900 CP, equipped with two Thermal Conductivity Detectors, responding to the difference in thermal conductivity between the carrier gas (Ar) and the sample components, with an error of ±3%.

Isotopic analysis on free gas (δ^13^C-CO_2_, δ^13^C-CH_4_, δD-CH_4_,) were performed at ISO4 S.n.c. Laboratory. The results were obtained by preparing the sample according to ref.^35^. Results are expressed in VSMOW and VPDB, following the International Atomic Energy Agency protocol.

### Statistical analysis

Standard statistical parameters were computed by Statistica 10.0 (StatSoft, Inc.). Normal probability plots (NPPs), were elaborated by Sinclair method to distinguish different populations and amore objective approach to statistical anomaly threshold estimation.

The normal probability plot of CO_2_ shows a data distribution characterized by six populations: i) background values, ranging from 0 to 1% v/v; ii) threshold anomaly, ranging from 1 and 2% v/v; iii) weak local anomaly, ranging from 2 to 3% v/v; iv) moderate anomalous values, up to 6% v/v; v) high anomalous values, ranging from 6 to 10% v/v; vi) outliers, with values higher than 10% v/v.

The NPP of H_2_ highlights a quite homogeneous distribution for values up to 10 ppm v/v, with background values from 0 to 1.8 ppm v/v, weak anomalies between 4 and 10 ppm v/v, moderate anomalies between 10 and 36 ppm v/v, high anomalies between 36 and 60 ppm v/v and outlier values over 60 ppm v/v.

For CH_4_, six populations were identified. The threshold anomaly is comprised between 0 and 1000 ppm v/v. The other populations are characterized by values ranging from 1000 ppm v/v to 4% v/v (local anomaly), from 4 to 10% v/v (weak anomaly), from 10 to 18% v/v (anomalous values), from 18 to 30% v/v (high anomalous values) and values over 30% v/v (outliers).

Data were displayed as 3D Surface maps were used to create a three-dimensional shaded rendering from a grid file. The height of the surface corresponds to the Z value of the associated grid node. These maps use colours to indicate the local orientation of the surface relative to a user-defined light source direction. The program Surfer 12.0 (Golden Software) determines the orientation of each grid cell and calculates reflectance of a point light source on the grid surface. The light position for all the maps is 135° for the horizontal angle and 45° for the vertical angle.

## Electronic supplementary material

Table S1, Table S2, Table S3
