# Supplementary material for: Learning from soil gas change and isotopic signatures during 2012 Emilia seismic sequence
Source: Sci Rep. 2017 Oct 27;7:14187. doi: 10.1038/s41598-017-14500-y (PMC5660157; doi:10.1038/s41598-017-14500-y)
Supplement: Supplementary file 1 — Table S1, Table S2, Table S3 [file 41598_2017_14500_MOESM1_ESM.doc]

**Learning from soil gas change and isotopic signatures during 2012 Emilia seismic sequence**

Alessandra Sciarra, Barbara Cantucci, Massimo Coltorti

**Table S1:** Descriptive statistics of soil gas concentration (37 sampling points) of H_2_, CH_4_, CO_2_ measured in the Medolla area from 2008 to 2015.

|  |  | H_2_  ppm v/v |  | CH_4_  % v/v |  | CO_2_  % v/v |
| --- | --- | --- | --- | --- | --- | --- |
| October 2008 | | | | | | |
| Mean |  | 1.63 |  | 3.58×10^-3^ |  | 0.13 |
| Median |  | 1.30 |  | 1.36×10^-4^ |  | 0.13 |
| Minimum |  | 0.87 |  | 0.34×10^-4^ |  | 0.02 |
| Maximum |  | 4.29 |  | 0.0485 |  | 0.28 |
| St. Dev. |  | 0.83 |  | 0.0115 |  | 0.06 |
| May 2012 | | | | | | |
| Mean |  | 9.36 |  | 6.46% |  | 5.43 |
| Median |  | 0.58 |  | 0.0515 |  | 4.80 |
| Minimum |  | 0.02 |  | 2.78×10^-4^ |  | 0.08 |
| Maximum |  | 89.25 |  | 40% |  | 13.46 |
| St. Dev. |  | 20.04 |  | 10.10 |  | 4.61 |
| September 2012 | | | | | | |
| Mean |  | 12.28 |  | 5.5% |  | 4.43 |
| Median |  | 0.73 |  | 3.1×10^-3^ |  | 2.75 |
| Minimum |  | 0.22 |  | 0.56 |  | 0.12 |
| Maximum |  | 147.59 |  | 39% |  | 12.25 |
| St. Dev. |  | 30.12 |  | 10.66 |  | 3.98 |
| June 2013 | | | | | | |
| Mean |  | 6.49 |  | 7.3% |  | 3.22 |
| Median |  | 1.41 |  | 0.050 |  | 1.73 |
| Minimum |  | 0.38 |  | 0.00 |  | 0.16 |
| Maximum |  | 50.48 |  | 55% |  | 12.75 |
| St. Dev. |  | 11.71 |  | 16.70 |  | 3.45 |
| June 2014 | | | | | | |
| Mean |  | 3.64 |  | 7.53% |  | 2.72 |
| Median |  | 0.95 |  | 7.51×10^-3^ |  | 1.79 |
| Minimum |  | 0.00 |  | 2.00×10^-4^ |  | 0.00 |
| Maximum |  | 37.24 |  | 84% |  | 11.01 |
| St. Dev. |  | 7.85 |  | 18.47 |  | 2.42 |
| May 2015 | | | | | | |
| Mean |  | 4.61 |  | 0.72% |  | 2.03 |
| Median |  | 0.95 |  | 1.01×10^-3^ |  | 1.06 |
| Minimum |  | 0.31 |  | 4.40×10^-4^ |  | 0.04 |
| Maximum |  | 54.52 |  | 8.9% |  | 6.32 |
| St. Dev. |  | 10.81 |  | 2.17 |  | 2.06 |

**Table S2:** Soil gas isotopic data (δ^13^C- CH_4_, δ^13^C- CO_2_, δD-CH_4_) collected from 2008 and 2015. For each survey, isotopic analyses were carried out on a subset of samples characterized by high anomalous concentrations.

| ID |  | δ^13^C-CH_4_ |  | δ^13^C-CO_2_ |  | δD-CH_4_ |
| --- | --- | --- | --- | --- | --- | --- |
| October 2008 | | | | | | |
| M3 |  | -29.86 |  | **-** |  | -92.26 |
| M20 |  | -25.88 |  | - |  | -106.44 |
| December 2008 | | | | | | |
| M3 |  | -66.89 |  | -48.59 |  | -182.7 |
| M17 |  | -49.90 |  | -21.98 |  | -137.39 |
| M20 |  | -68.07 |  | -58.18 |  | -187.95 |
| M23 |  | -25.8 |  | -10.96 |  | -125.44 |
| M28 |  | -39.13 |  | -42.15 |  | -126.13 |
| May 2012 | | | | | | |
| M3 |  | -59.08 |  | -61.50 |  | -163.07 |
| M20 |  | -59.02 |  | -58.49 |  | -168.70 |
| M36 |  | -61.75 |  | -70.01 |  | -172.71 |
| September 2012 | | | | | | |
| M3 |  | -57.30 |  | -60.75 |  | -155.30 |
| M14 |  | -64.34 |  | -66.54 |  | -174.15 |
| M14_4 |  | -62.49 |  | -65.89 |  | -72.83 |
| M20 |  | -59.03 |  | -57.77 |  | -161.59 |
| M22 |  | -21.51 |  | -20.31 |  | -100.21 |
| M35 |  | -61.45 |  | -57.24 |  | -167.25 |
| M37 |  | -32.58 |  | -37.04 |  | -96.99 |
| June 2013 | | | | | | |
| M3 |  | -60.49 |  | -51.47 |  | -154.5 |
| M14 |  | -70.85 |  | -58.67 |  | -187.8 |
| M36 |  | -56.80 |  | -55.89 |  | -167.4 |
| M35 |  | -66.36 |  | -45.38 |  | -172.7 |
| M20_5 |  | -63.76 |  | -56.46 |  | -177.2 |
| M14_4 |  | -76.01 |  | -61.98 |  | -155.8 |
| M20 |  | -53.85 |  | -57.38 |  | -163.0 |
| M20_4 |  | -66.48 |  | -54.35 |  | -174.5 |
| June 2014 | | | | | | |
| M3 |  | -58.49 |  | -55.47 |  | -160.5 |
| M14 |  | -78.85 |  | -56.78 |  | -178.8 |
| M36 |  | -56.80 |  | -52.79 |  | -167.4 |
| M35 |  | -61.36 |  | -55.38 |  | -171.7 |
| M20 |  | -65.85 |  | -53.28 |  | -173.0 |
| M20_4 |  | -68.48 |  | -56.35 |  | -176.5 |
| May 2015 | | | | | | |
| M3 |  | -55.49 |  | -51.47 |  | -170.5 |
| M14 |  | -68.85 |  | -57.98 |  | -178.8 |
| M36 |  | -56.80 |  | -55.89 |  | -167.4 |
| M35 |  | -61.36 |  | -54.68 |  | -171.7 |
| M20 |  | -55.85 |  | -50.24 |  | -153.0 |
| M37 |  | -35.85 |  | -38.45 |  | -100.7 |

**Table S3:** Soil gas isotopic data (δ^13^C-CH_4_, δ^13^C-CO_2_, δD-CH_4_) collected in 2015 for M20 point at different depth (0.20, 1.0 and 2.5 m).

| **ID** | **Depht (m)** | δ **^13^C(CO_2_)**  vs VPDB | δ **^18^O(CO_2_)** vs VPDB | δ**^13^C(CH_4_)** vs VPDB | δD **(CH_4_)** vs VSMOW |
| --- | --- | --- | --- | --- | --- |
| M20 | 0.20 | -65.51 | -8.57 | -62.34 | -156.4 |
| M20 | 1.00 | -12.12 | -3.39 | -68.51 | -177.8 |
| M20 | 2.50 | -1.51 | -4.16 | -34.11 | -89.72 |
